# β2* nicotinic acetylcholine receptor subtypes mediate nicotine-induced enhancement of Pavlovian conditioned responding to an alcohol cue

**DOI:** 10.3389/fnbeh.2022.1004368

**Published:** 2022-10-12

**Authors:** Jean-Marie Maddux, Leslie Gonzales, Nathaniel P. Kregar

**Affiliations:** ^1^Department of Psychology, Lake Forest College, Lake Forest, IL, United States; ^2^Neuroscience Program, Lake Forest College, Lake Forest, IL, United States

**Keywords:** nicotine, ethanol, Pavlovian conditioned approach, goal-tracking, nicotinic acetylcholine receptor, associative learning

## Abstract

Nicotine enhances Pavlovian conditioned responses to reward-associated cues. We investigated through which nicotinic acetylcholine receptor (nAChR) subtypes nicotine acts to produce this behavioral effect to an alcohol-associated cue. Male Long-Evans rats with freely available food and water were first accustomed to drinking 15% ethanol in their home cages using an intermittent access, two-bottle choice procedure. Then the rats were given 15 Pavlovian conditioning sessions in which a 15-s audiovisual conditioned stimulus (CS) predicted the delivery of 0.2 ml of ethanol, the unconditioned stimulus (US). Each session contained 12 CS-US trials. A control group received explicitly unpaired presentations of the CS and US. We measured Pavlovian conditioned approach to the site of US delivery during presentations of the CS, accounting for pre-CS baseline activity. Before each conditioning session, rats were injected subcutaneously with nicotine (0.4 mg/kg) or saline (1 ml/kg). During nAChR antagonist test sessions, rats were first injected systemically with the β2*-selective nAChR antagonist dihydro-beta-erythroidine (DHβE; 3 mg/kg) or the α7-selective nAChR antagonist methyllycaconitine (MLA; 6 mg/kg), followed by their assigned nicotine or saline injection before assessing their conditioned response to the alcohol-associated cue. Consistent with previous reports, nicotine enhanced the Pavlovian conditioned response to the alcohol-paired cue. DHβE attenuated this enhancement, whereas MLA did not. These results suggest that nicotine acts via β2*, but not α7, nAChRs to amplify Pavlovian conditioned responding to an alcohol cue. These findings contribute to a growing literature that identifies nAChRs as potential targets for pharmacological treatment of co-morbid alcohol and tobacco use disorders.

## Introduction

Global statistics on substance use consistently identify tobacco and alcohol use as leading causes of disability and premature mortality (Peacock et al., 2018). Nicotine, the main psychoactive ingredient in tobacco, and alcohol are often co-used, and research indicates that each substance has the ability to potentiate craving for, and self-administration of, the other, in a reciprocal fashion (Verplaetse and McKee, 2017). Nicotinic acetylcholine receptors (nAChRs) provide a promising pharmacological target in the potential treatment of alcohol use disorder and/or nicotine dependence, given that nAChRs are a shared biological target of nicotine and ethanol (Klenowski and Tapper, 2018).

Environmental stimuli associated with drug effects can gain powerful control over behavior through Pavlovian (classical) conditioning (Rohsenow et al., 1990; Glautier and Drummond, 1994; Field and Duka, 2002; Witteman et al., 2015). In this framework, a sensory stimulus that reliably predicts the pharmacological effects of a drug is considered a conditioned stimulus (CS) that becomes associated with the unconditioned stimulus (US) of the drug’s effects. The CS can then trigger a variety of conditioned responses (CR) that may fuel further drug use, such as craving, approach, and seeking (engaging in behaviors to attempt to procure the drug). Indeed, addiction can be viewed as a type of learned behavior (Hägele et al., 2014; Heinz et al., 2019). As such, controlled laboratory studies using simple Pavlovian conditioning procedures can offer insight into behavioral processes that contribute to drug use. Multiple studies have shown that alcohol-predictive cues trigger physiological (such as changes in salivation, heart rate, or skin conductance), psychological (such as craving), and behavioral (such as approach, seeking, or orienting) conditioned responses in humans (Pomerleau et al., 1983; Monti et al., 1987; Staiger and White, 1991; Greeley et al., 1993; Collins and Brandon, 2002; Field and Duka, 2002; Field et al., 2005, 2008) and rodents (Bachteler et al., 2005; Cunningham and Patel, 2007; Krank et al., 2008; Villaruel and Chaudhri, 2016; Alarcón and Delamater, 2019; Cofresí et al., 2019; Loney et al., 2019; Lamb et al., 2020; Valyear and Chaudhri, 2020).

Given the potentiating effects of nicotine on alcohol craving, seeking, and consumption (Verplaetse and McKee, 2017), a relevant question that has begun to be addressed concerns the extent to which nicotine affects the conditioned response to an alcohol-paired cue (Maddux and Chaudhri, 2017; Loney et al., 2019; Angelyn et al., 2021). Maddux and Chaudhri (2017) found that nicotine increased Pavlovian conditioned approach to the site of reward delivery (goal-tracking), in response to an alcohol cue (which was an audiovisual compound), but conditioned approach to the reward-predictive cue itself (sign-tracking) was not measured in that study. Loney et al. (2019) and Angelyn et al. (2021) demonstrated that nicotine enhanced goal-tracking, but not sign-tracking, in response to an alcohol cue (which was a retractable lever). In the present study, we wished to directly extend the findings of our earlier work, hence we chose to use the same behavioral paradigm as that of Maddux and Chaudhri (2017). In addition to demonstrating that nicotine increased Pavlovian conditioned approach in response to an alcohol cue, that report also showed that the non-selective nAChR antagonist mecamylamine blocked this effect (Maddux and Chaudhri, 2017). This observation indicates that the nicotine-induced enhancement of the Pavlovian conditioned response is mediated by nAChRs. Here, we wanted to determine which nAChR subtypes are responsible for the nicotine-induced increase of Pavlovian conditioned approach triggered by an alcohol cue. We did this by conducting test sessions using a selective α7 nAChR antagonist, methyllycaconitine (MLA), and a selective α4β2 nAChR antagonist, dihydro-beta-erythroidine (DHβE). These particular nAChR subtypes were targeted because they are the most abundant nAChR subtypes in the brain, with distribution in brain areas that comprise neural reward circuitry (Feduccia et al., 2012; Hendrickson et al., 2013). Moreover, α4β2 nAChRs specifically have been implicated in mediating nicotine reward or reinforcement across a range of behavioral paradigms (Picciotto et al., 1998; Brunzell et al., 2006; Kenny and Markou, 2006; Walters et al., 2006; Liu et al., 2007; Guy and Fletcher, 2013; Tabbara and Fletcher, 2019), many of which also demonstrated a lack of involvement of α7 nAChRs in these same behaviors (Walters et al., 2006; Liu et al., 2007; Guy and Fletcher, 2013). Therefore, we expected DHβE, but not MLA, to attenuate the nicotine-induced enhancement of goal-tracking to a CS for alcohol. DHβE is frequently classified as an α4β2 nAChR antagonist because this subtype is highly sensitive to DHβE (Khiroug et al., 2004) and α4β2 nAChRs are the most abundant nAChRs to which nicotine binds with high affinity in the brain (Picciotto et al., 2001). However, DHβE has the ability to bind to other nAChR subtypes, but with less potency (Harvey et al., 1996; Verbitsky et al., 2000; Papke et al., 2008; Capelli et al., 2011). Moving forward in this article, we adopt the nomenclature β2* nAChRs to refer to those receptors to which DHβE binds with high selectivity, where the * indicates other subunits that coassemble with β2 to form a pentameric nAChR complex (Lukas et al., 1999).

## Materials and Methods

### Subjects

Forty-six male Long-Evans rats purchased from Envigo (Barrier 202B; Indianapolis, IN, USA) were used in this experiment. The rats weighed 220–240 g and were approximately 8 weeks old upon arrival in the lab. All animals were individually housed and maintained on a 12-h light-dark cycle (lights on at 8:00 AM) in a temperature (21°C; acceptable range: 20°C–26°C) and humidity (46%; acceptable range: 30%–70%) controlled room. Each polycarbonate home cage (item R20, Ancare, Bellmore, NY, USA) measured 48.3 × 26.7 × 20.3 cm and contained wood shavings bedding (item 11003292, Charles River Laboratories, Wilmington, MA, USA). Rats did not receive enrichment objects in their home cages. All experimental procedures took place during the light phase, between 10 AM and 6 PM. Each cohort of rats was trained and tested at a consistent time of day. Rats had unrestricted access to food and water throughout all experimental procedures, which were approved by the Lake Forest College Institutional Animal Care and Use Committee (IACUC). Rats were handled and weighed daily for a minimum of 7 days before experimental procedures began.

### Home cage ethanol access

Rats were acclimated to the taste and pharmacological effects of 15% ethanol using a 24-h, intermittent access, 2-bottle choice procedure in the home cage (Wise, 1973; Simms et al., 2008). Although the initial reports by Wise (1973) and Simms et al. (2008) used 20% ethanol, several research groups have successfully used the intermittent access, 2-bottle choice procedure with 15% ethanol (Cofresí et al., 2018, 2019; Angelyn et al., 2021). Ethanol (15% v/v) was prepared by diluting 95% ethanol in tap water. Rats were given access to two separate bottles on the lid of the home cage; one contained 15% ethanol and the other contained water. After a 24-h access period, the ethanol bottle was removed. Hence, rats had 24-h access to both ethanol and water, followed by 24-h access to only water in a given session. This procedure was repeated for a total of 12 ethanol exposure sessions. Positions of the ethanol and water bottles on the cage lid (left vs. right) were counterbalanced across sessions to discourage the development of a side preference. Bottles were weighed before placement onto the home cage and after the 24-h access period; subtraction of bottle weights (weight on − weight off) allowed us to determine both water and ethanol consumption. Spillage and evaporation were accounted for by subtracting the amount of water or ethanol lost from bottles placed on empty cages from the average consumption of each solution, respectively, during the corresponding session. In the rare instance that a rat drank less than the spillage amount, consumption was coded as zero for that session. Rats that consumed less than 1.0 g/kg/24 h of ethanol averaged across the two previous sessions were provided a sweetened ethanol solution (15% ethanol with 2% sucrose, w/v) to encourage ethanol intake. This measure was instituted starting in either sessions 6, 7, or 8. Exposure to sweetened ethanol was minimized, both in terms of number of rats receiving it (see Results section, Exclusion criteria and final sample sizes) and duration of receipt. No rat received sweetened ethanol for more than two consecutive sessions. Any rat that did receive sweetened ethanol was returned to regular, unsweetened 15% ethanol by session 9 at the latest. Dependent measures for the home cage ethanol access phase were ethanol intake (grams of ethanol consumed per kg of rat body weight, g/kg) and ethanol preference (grams of ethanol solution consumed as a percentage of total fluid consumption).

### Apparatus

Near the end of the home cage 2-bottle choice procedure, rats were habituated to the behavior testing room on two separate days (after ethanol bottles were removed from the home cage lids at the conclusion of sessions 11 and 12). Following the 12 sessions of home cage ethanol access, rats were habituated to the testing chambers in a single, 20-min session. The apparatus consisted of eight identical modular behavioral testing chambers (ENV-007) obtained from Med Associates Inc. (St. Albans, VT, USA). Each chamber was enclosed in a ventilated, sound-attenuating medium density fiberboard (MDF) cubicle (ENV-018MD). Each chamber had a clear polycarbonate ceiling, front and back wall, and stainless steel side walls. A stainless steel waste pan (ENV-007A3) lined with aspen chip bedding was placed below each chamber’s stainless steel grid rod floor (ENV-005). The chambers were configured similarly to those previously described in Maddux and Chaudhri (2017), with the following difference: ethanol was delivered into a small, circular fluid receptacle that was part of a dual pellet/liquid cup (ENV-202RMA). The food pellet feature was never used in this experiment; only liquid ethanol was delivered. The lower right wall of each chamber contained a centrally located alcove in which the liquid receptacle was housed. Ethanol was delivered into the liquid receptacle, which was located on the right side of the alcove, via polyvinyl chloride (PVC) tubing connected to a 20-ml syringe that was affixed to a syringe pump (PHM-100; 3.33 rpm) located outside of the sound-attenuating cubicle. Entries into the alcove were measured by disruption of an infrared photobeam (ENV-254-CB) positioned across its opening. Two white incandescent stimulus lights (ENV-221M; 28 V DC; 100 mA) were mounted on the right chamber wall, one on each side of the fluid receptacle alcove, 10.7 cm above the chamber floor. Two retractable levers (ENV-112CM) were also located on this wall, but they were not used and remained retracted throughout the entire experiment. The upper left wall of each chamber included a centrally located white incandescent house light (ENV-215M; 28 V DC; 100 mA) and, to the left of the house light, an audio speaker (ENV-224AM) connected to a white noise generator (ENV-225S; 80–85 dB) located on the exterior of the chamber (but within the sound-attenuating cubicle). A PC desktop computer running Med-PC V software (Med Associates Inc., St. Albans, VT, USA) for Windows controlled experimental events and recorded entries into the liquid cup.

### Habituation to procedure

During the chamber habituation session, the house light was illuminated for 20 min and entries into the (empty) liquid cup were recorded. To habituate the rats to the systemic injection procedure, all rats received a subcutaneous injection of sterile 0.9% saline (injection volume 1.0 ml/kg) 10 min before the chamber habituation session.

### Pavlovian conditioning

Two days after the habituation session, Pavlovian conditioning sessions began. Conditioning sessions were conducted on an alternating schedule (sessions took place on Monday, Wednesday, and Friday of each week) in order to maintain intermittent access to ethanol and promote ethanol consumption (in the testing chambers). Each session started with a 2-min delay period during which liquid cup entries were counted. At the end of the delay period, the house light turned on and remained illuminated for the duration of the session (63 min, on average). Within each conditioning session, 12 trials were presented to the rats. For rats in the paired group, a trial consisted of the presentation of an audiovisual CS (simultaneous onset of white noise and two white stimulus lights) coupled with delivery of 0.2 ml of 15% ethanol, which served as the US, to the liquid cup. The CS was 15 s in duration, the first 9 s of which were solely the CS; during the last 6 s of the CS, the fluid pump was activated such that ethanol US was delivered to the liquid cup. Hence, for rats in the paired group, the CS and US overlapped in time for the final 6 s of the CS, and then co-terminated. For rats in the unpaired control group, a trial consisted of these same CS and US events, but in an explicitly unpaired fashion. This was arranged by delivering the ethanol US midway through the interval between two CS presentations, such that the US was temporally distant from the CS. For both training groups, the CS was delivered on a variable time 270-s schedule (inter-CS intervals were 150, 270, and 390 s. Both paired and unpaired groups received the same number of CS and US presentations, and CS presentations occurred at the same time for both groups. Both groups received 2.4 ml of ethanol per session (distributed across 12 trials).

### Drugs

Ten minutes prior to each Pavlovian conditioning session, rats were injected subcutaneously with either nicotine (0.4 mg/kg; dose expressed as free base) or sterile 0.9% saline (injection volume 1.0 ml/kg for both nicotine and saline groups), according to their assigned drug group. Nicotine solution was prepared by dissolving (-)-nicotine ditartrate salt (Tocris Bioscience; catalog number 3546) in sterile 0.9% saline and adjusting the pH to 7.0–7.2 using liquid sodium hydroxide. Rats received 15 Pavlovian conditioning sessions, after which they were tested with the nAChR antagonists DHβE (3.0 mg/kg; 1.0 ml/kg) and MLA (6.0 mg/kg; 1.0 ml/kg). Both DHβE (catalog number 2349) and MLA (catalog number 1029) were obtained from Tocris Bioscience, and dissolved in sterile 0.9% saline.

### nAChR antagonist test sessions

Test sessions were identical to the previous conditioning sessions, with the exception that now rats received two sets of injections before each test session. The first injection was either saline or a nAChR antagonist (either DHβE or MLA), and the second injection was either saline or nicotine, as per the previous assigned drug group. Twenty minutes elapsed between the first injection and the second injection. Each rat was tested four times, in a counterbalanced, within-subject design. For example, the first set of two tests for a given rat could be a DHβE test and a corresponding saline test and the second set of two tests for the same rat would then be an MLA test and a corresponding saline test (order counterbalanced across rats). To acclimate rats to the double-injection procedure before testing, all rats received a saline injection before receiving their usual assigned drug group injection (nicotine or saline) in session 15 of Pavlovian conditioning. One regular (non-test) Pavlovian conditioning session intervened between the two rounds of testing, which served as a re-training day. This re-training session maintained the double-injection procedure, with all rats first receiving a saline injection before receiving their usual assigned drug group injection (nicotine or saline).

**Figure 1:**
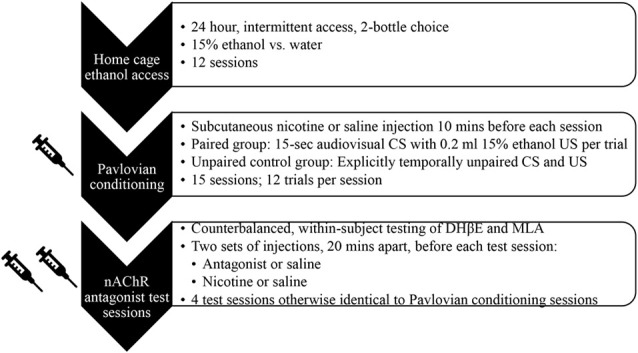
Schematic diagram of experimental timeline, with key procedural details of each phase.

A schematic diagram of the timeline of all phases of the experiment is provided in Figure 1.

### Statistical analyses

The main dependent measure for Pavlovian conditioning sessions and nAChR antagonist test sessions was the number of liquid cup entries made during the first 9-s of the CS minus the number of liquid cup entries made during a corresponding 9-s pre-CS baseline interval; this yielded a normalized CS liquid cup entry measure. Greater responding during the CS interval relative to the pre-CS interval is indicative of conditioned behavior. Analyses using the normalized CS liquid cup measure and analyses using the separate CS and pre-CS measures yielded results that lead to identical conclusions (see Supplementary Table 1 for analysis including the CS and pre-CS intervals as a within-subject factor), and so we report the results of the simplified normalized CS liquid cup entry measure, however, we show both pre-CS and CS responding in our figures for transparency. We restricted our analysis to the first 9 s of the 15 s CS because this allows for a measure of conditioned responding that is not confounded by the presence of ethanol in the liquid cup (as occurred during the last 6 s of the CS in the paired group). Liquid cup entries made during the 2-min delay period at the beginning of each session are also reported (see Supplementary Figure 1), as these data serve as a measure of behavior that is separate from liquid cup entries made during the training session per se (once stimulus events are presented) and thus allows for assessment of possible nicotine-induced locomotor effects on liquid cup entry behavior. Total liquid cup entries in each session are also provided (see Supplementary Figures 2, 3). Data were analyzed using repeated-measure ANOVA in SPSS (IBM SPSS Statistics Version 26) and Statistica (Version 5, StatSoft). The factors used in each analysis are described in the results that follow. All analyses used the statistical significance level of *α* = 0.05. The Huyhn-Feldt correction was applied for violations of sphericity as detected by Mauchly’s test. Significant interactions were pursued with follow-up *post-hoc* multiple comparison testing using Tukey’s Honestly Significant Difference (HSD) test.

In an effort to characterize any potential relationship between home cage ethanol consumption and the conditioned response during Pavlovian conditioning sessions, the Pearson correlation coefficient was also used. Ethanol consumption was averaged across the last two sessions of home cage ethanol access. Similarly, CS-evoked liquid cup entries during the last two Pavlovian conditioning sessions were averaged for this analysis. These measures were then subjected to Pearson’s correlation, split by behavior group and drug group. For the correlational analysis, a normalized CS liquid cup entry measure was also used, which takes pre-CS baseline responding into account, by subtracting the number of liquid cup entries made during the pre-CS interval from the number of liquid cup entries made during the CS interval. This allowed for ease of visual presentation in the scatterplot.

## Results

### Home cage ethanol access

#### Exclusion criteria and final sample sizes

Six rats were excluded from the study based on low ethanol intake during the home cage ethanol consumption phase. All included rats had greater than 0.5 g/kg/24 h ethanol intake averaged across the last two sessions of home cage ethanol access. Final sample sizes for each group are as follows: paired nicotine, *n* = 12; paired saline, *n* = 12; unpaired nicotine, *n* = 8; unpaired saline, *n* = 8. Data from these 40 rats are analyzed and reported. Of these 40 rats, eight rats received the sweetened ethanol solution (15% ethanol with 2% sucrose, w/v) for two sessions during the middle of the home cage ethanol consumption phase. These eight rats were distributed equally across the nicotine and saline drug treatment groups. A separate analysis for the Pavlovian conditioning sessions as well as the nAChR antagonist test sessions that included exposure to sweetened ethanol during the home cage consumption phase as a between-subjects factor revealed no significant effect of, nor any interactions with, previous experience with sweetened ethanol, all *F* < 2.562, all *p* > 0.119.

#### Home cage ethanol consumption and preference

**Figure 2:**
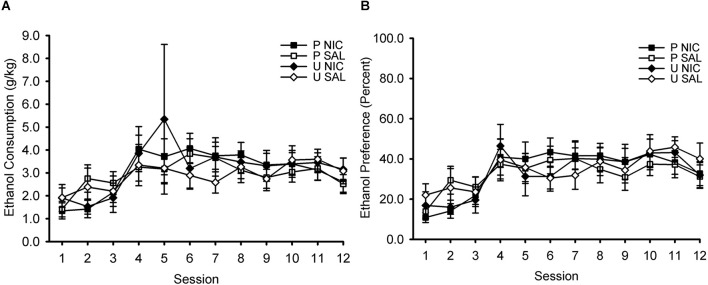
Ethanol consumption **(A)** and ethanol preference **(B)** increased across sessions of intermittent access to 15% ethanol and water in the home cage. Data represent the mean (± SEM) values for each session. After this phase, rats were divided into four groups for Pavlovian conditioning training, in which they received either paired (P) or unpaired (U) trials of an audiovisual conditioned stimulus (CS) and ethanol unconditioned stimulus (US) and pre-session injections of either nicotine (NIC) or saline (SAL). This design resulted in the following four groups: P NIC (black squares), P SAL (white squares), U NIC (black diamonds), and U SAL (white diamonds). There were no differences in ethanol consumption or ethanol preference between these four training groups.

During the home cage 2-bottle choice intermittent ethanol access phase of the experiment, ethanol intake (measured in g/kg; Figure 2A) and ethanol preference (percentage; Figure 2B) increased across sessions. ANOVA with the within-subject factor of session (1–12) and the between-subject factors of behavior group (paired, unpaired) and drug group (nicotine, saline) revealed only a significant main effect of session on ethanol intake, *F*_(11,396)_ = 5.90, *p* < 0.001, and ethanol preference, *F*_(11,396)_ = 11.81, *p* < 0.001. No other effects or interactions were significant, all *p* > 0.05. Note that the manipulations of behavior group and drug group had not yet occurred at this phase of the experiment. These factors are included in this analysis to ensure that assignment to behavior group and drug group was properly counterbalanced with respect to ethanol intake and ethanol preference.

### Pavlovian conditioned approach

**Figure 3:**
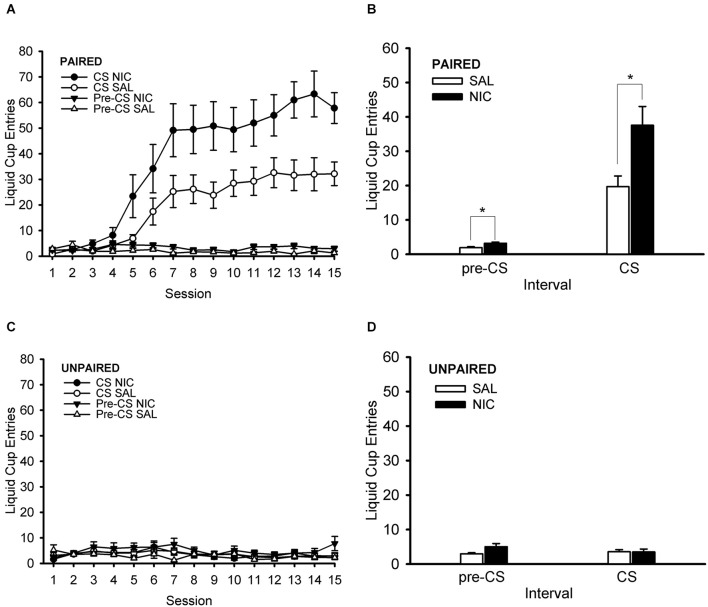
Nicotine administered systemically before each Pavlovian conditioning session enhanced the Pavlovian conditioned response to an alcohol cue. Data represent the mean (± SEM) number of liquid cup entries per session (summed across 12 trials per session) during the first 9 s of the CS and during a 9-s pre-CS interval for the paired group **(A)** and the unpaired group **(C)**. The mean (± SEM) number of liquid cup entries averaged across all 15 sessions of Pavlovian conditioning during the CS and pre-CS intervals for the paired group **(B)** and unpaired group **(D)** is also displayed. Black symbols or bars denote nicotine (NIC) treatment and white symbols or bars denote saline (SAL) treatment. *p < 0.05, NIC > SAL.

During the Pavlovian conditioning phase of the experiment, rats treated pre-session with nicotine showed a greater conditioned response than their saline-treated counterparts (Figure 3). Importantly, this effect occurred in the paired training group (Figures 3A,B) but not in the unpaired (control) training group (Figures 3C,D). The unpaired training group did not show evidence of conditioned responding, thereby validating its use as a control group. ANOVA with the within-subject factor of session (1–15) and the between-subjects factors of behavior group (paired, unpaired) and drug group (nicotine, saline) showed main effects of session and behavior group, *F*_(14,504)_ = 20.261, *p* < 0.001 and *F*_(1,36)_ = 48.039, *p* < 0.001, respectively. Significant interactions in this analysis were session × behavior group and behavior group × drug group, *F*_(14,504)_ = 20.615, *p* < 0.001 and *F*_(1,36)_ = 6.008, *p* = 0.019, respectively. Follow-up *post hoc* multiple comparison testing using Tukey’s HSD test showed that the paired group had greater normalized CS liquid cup entries than the unpaired group starting on Session 6 and continuing through the end of Pavlovian training on Session 15, all *p* < 0.001. More importantly, *post-hoc* Tukey HSD test that examined the behavior group × drug group interaction showed that the paired nicotine group had a higher conditioned response than each of the other three groups (paired saline, unpaired nicotine, and unpaired saline), all *p* < 0.001. The paired saline group had higher conditioned responding than each of the two unpaired groups (unpaired nicotine and unpaired saline), all *p* < 0.016. The two unpaired groups (unpaired nicotine and unpaired saline) did not differ from each other, *p* = 0.983. In summary, rats in the paired group showed evidence of conditioned responding that grew across sessions and was enhanced by nicotine treatment.

### Correlation of home cage ethanol consumption with Pavlovian conditioned approach

**Figure 4:**
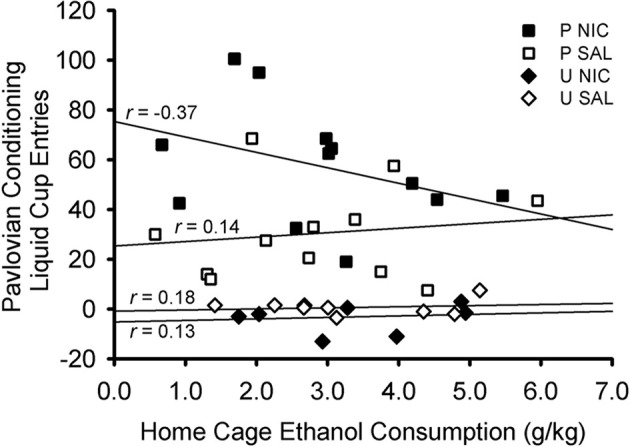
Scatterplot showing the non-significant correlations between terminal levels of home cage ethanol consumption and Pavlovian conditioned responding. Ethanol consumption was averaged across the last two sessions of home cage ethanol access. Liquid cup entries made in response to the CS (with pre-CS baseline responding subtracted) were averaged across the last two sessions of Pavlovian conditioning. Each symbol represents an individual rat. Symbol shape and color indicate behavioral training group and drug group: black squares denote rats in the paired nicotine group, white squares denote rats in the paired saline group, black diamonds denote rats in the unpaired nicotine group, and white diamonds denote rats in the unpaired saline group. Pearson’s *r* values are indicated for each treatment group.

There was no relationship between terminal levels (average of last two sessions) of home cage ethanol consumption and terminal levels (average of last two sessions) of Pavlovian conditioned responding (Figure 4). Separate Pearson’s correlations were conducted for each treatment group: paired nicotine, *r*_(10)_ = −0.371, *p* = 0.235; paired saline: *r*_(10)_ = 0.144, *p* = 0.655; unpaired nicotine: *r*_(6)_ = 0.126, *p* = 0.766; unpaired saline: *r*_(6)_ = 0.177, *p* = 0.674. Moreover, no relationship existed when groups were considered by behavioral training group (paired: *r*_(22)_ = −0.110, *p* = 0.609; unpaired: *r*_(14)_ = 0.134, *p* = 0.622) or drug group (nicotine: *r*_(18)_ = −0.291, *p* = 0.214; saline: *r*_(18)_ = −0.034, *p* = 0.885).

### nAChR antagonist test sessions

#### Pavlovian retraining

ANOVA comparing the Pavlovian retraining session that intervened between the two rounds of nAChR antagonist testing with the last day of Pavlovian training (session 15) that preceded any testing showed no effect of session, nor any interactions with session, all *F* < 2.062, *p* > 0.160. This indicates that performance was not different between these two sessions and validates the use of one retraining session as sufficient to return to baseline.

#### DHβE test session

**Figure 5:**
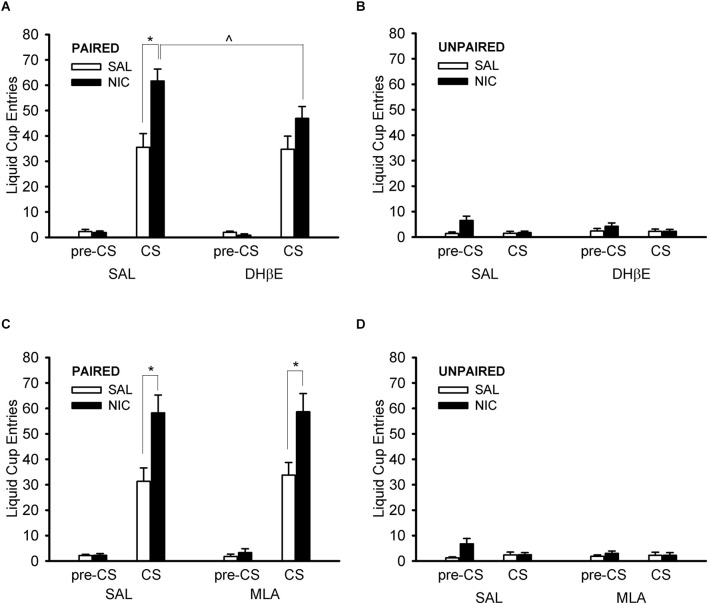
Pre-treatment with the β2* nAChR antagonist dihydro-beta-erythroidine (DHβE) attenuated the enhancement of the Pavlovian conditioned response to an alcohol cue caused by nicotine **(A)**, but the α7 nAChR antagonist methyllycaconitine (MLA) had no such effect **(C)**. Data represent the mean (± SEM) number of liquid cup entries during the first 9 s of the CS and during a 9-s pre-CS interval for the paired group **(A,C)** and the unpaired group **(B,D)** in the within-subject nAChR antagonist test sessions. Black bars denote nicotine (NIC) treatment and white bars denote saline (SAL) treatment. *p < 0.05, SAL/NIC > SAL/SAL and MLA/NIC > MLA/SAL. ^∧^*p* < 0.05, DHβE/NIC < SAL/NIC.

Treatment with DHβE prevented the nicotine-induced enhancement of CS liquid cup entries in the paired group (Figure 5A). ANOVA with the within-subject factor of nAChR antagonist treatment (DHβE, saline) and the between-subjects factors of behavior group (paired, unpaired) and drug group (nicotine, saline) showed a main effect of behavior group, *F*_(1,36)_ = 116.406, *p* < 0.001, and several significant interactions: behavior group × drug group, antagonist treatment × behavior group, and antagonist treatment × behavior group × drug group, *F*_(1,36)_ = 7.921, *p* = 0.008, *F*_(1,36)_ = 7.972, *p* = 0.008, and *F*_(1,36)_ = 7.501, *p* = 0.010, respectively. Follow-up *post hoc* multiple comparison testing of the 3-way interaction using Tukey’s HSD test showed that the paired nicotine group (Figure 5A) responded less when treated with DHβE compared to saline, *p* = 0.0003, but the paired saline group (Figure 5A), the unpaired nicotine group (Figure 5B), and the unpaired saline group (Figure 5B) did not respond differently when treated with DHβE compared to saline, all *p* > 0.989. Importantly, the nAChR antagonist DHβE reduced conditioned responding elicited by the CS in the nicotine-treated rats of the paired group, suggesting that the nicotine-induced enhancement of Pavlovian conditioned responding to an alcohol cue is mediated via β2* nAChRs.

#### DHβE test session: trial-by-trial analysis

**Figure 6:**
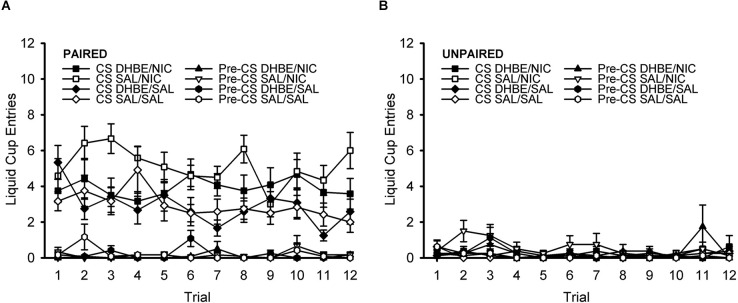
Pavlovian conditioned response to an alcohol cue during the DHβE test session shown on a trial-by-trial basis. The attenuation of the nicotine-induced enhancement in the Pavlovian conditioned response to an alcohol cue by the β2* nAChR antagonist dihydro-beta-erythroidine (DHβE) did not change significantly across the course of the test session. Data represent the mean (± SEM) number of liquid cup entries during the first 9 s of the CS and during a 9-s pre-CS interval for the paired group **(A)** and the unpaired group **(B)**. Black symbols represent DHβE treatment and white symbols represent saline treatment. In the legend, the abbreviation before the slash identifies the antagonist treatment (DHβE or saline) and the abbreviation after the slash identifies the drug group (nicotine or saline).

To further characterize the response across the course of the DHβE test session, we next analyzed the response on a trial-by-trial basis (Figures 6A,B). ANOVA with the within-subject factors of trial (1–12) and nAChR antagonist treatment (DHβE, saline) and the between-subject factors of behavior group (paired, unpaired) and drug group (nicotine, saline) showed no effect of trial, nor any interactions with trial, all *F* < 1.361, *p* > 0.191. This indicates that responding did not change significantly across the course of the test session. Rather, this analysis simply reiterated all the same effects and interactions present in the original omnibus ANOVA of the DHβE test session described in the previous DHβE test session section.

#### MLA test session

Treatment with MLA had no effect on liquid cup entries at all (Figures 5C,D). ANOVA with the within-subject factor of nAChR antagonist treatment (MLA, saline) and the between-subjects factors of behavior group (paired, unpaired) and drug group (nicotine, saline) showed main effects of behavior group and drug group, *F*_(1,36)_ = 70.171, *p* < 0.001 and *F*_(1,36)_ = 4.319, *p* = 0.045, respectively, and an interaction of behavior group × drug group, *F*_(1,36)_ = 7.274, *p* = 0.011. There were no effects of, or interactions with, the factor of nAChR antagonist treatment in the MLA test session, all *F* < 1.532, all *p* > 0.224. Importantly, the analysis of the MLA test session data showed that treatment with the α7 nAChR antagonist MLA did not change the nicotine-induced enhancement of the Pavlovian conditioned response to an alcohol cue, suggesting that this observation is not mediated by α7 nAChRs.

## Discussion

We found that nicotine administered before each Pavlovian conditioning session increased conditioned responding to an alcohol cue, an effect that replicates our previous work, as well as work from other laboratories (Maddux and Chaudhri, 2017; Loney et al., 2019; Angelyn et al., 2021). The novel contribution reported in this study is that this effect is mediated by β2* nAChRs, but not α7 nAChRs. This conclusion is supported by the observation of a reduced conditioned response to the alcohol cue in the nicotine-treated group when tested with DHβE, a selective α4β2 nAChR antagonist. By contrast, no such reduction was seen when the rats were tested with MLA, a selective α7 nAChR antagonist.

Similar dissociations in behavior under these nAChR antagonists have been reported in the literature across a variety of behavioral paradigms (Davis and Gould, 2006; Walters et al., 2006; Liu et al., 2007; Struthers et al., 2009; Guy and Fletcher, 2013). In general, these findings converge on the conclusion that β2* nAChRs are critically involved in nicotine’s enhancement of conditioned behavior, as the effects of nicotine are attenuated by DHβE but not MLA. Notably, these effects are not limited to appetitive conditioning, but also extend to aversive conditioning (Davis and Gould, 2006). The broad range of behavioral paradigms in which these effects are observed suggests robust involvement of the cholinergic system in reward-related learning and/or performance of conditioned responses. For instance, nicotine has been shown to support conditioned place preference (Walters et al., 2006), serve as an interoceptive CS (Struthers et al., 2009), exert reinforcement enhancing effects in operant responding (Liu et al., 2007), enhance contextual fear conditioning (Davis and Gould, 2006), and increase operant responding for a conditioned reinforcer (Guy and Fletcher, 2013). In each of these cases, the effects of nicotine were mediated by β2* nAChRs. We add to this growing body of literature by showing that nicotine increases Pavlovian conditioned responding to an alcohol cue, and that this effect is also mediated by β2* nAChRs.

We tested only one dose of each antagonist, and we acknowledge this as a limitation of the current study. However, the dose of DHβE used here was chosen based on published reports showing behavioral effects at this dose (Stolerman et al., 1997; Struthers et al., 2009; Guy and Fletcher, 2013). Struthers et al. (2009) reported full antagonism (to saline levels) of conditioned responding evoked by the interoceptive stimulus effects of nicotine with DHβE at a dose of 10 mg/kg and partial antagonism (lower than nicotine but still higher than saline) of conditioned responding at a dose of 3 mg/kg. However, Guy and Fletcher (2013) reported that DHβE at a dose of 3 mg/kg completely blocked (down to saline levels) the nicotine-induced enhancement of conditioned reinforcement. Thus, it seems likely that the degree of antagonism of a particular response at a given dose of DHβE depends upon the specific behavioral paradigm. Indeed, Stolerman et al. (1997) reported that DHβE antagonized the locomotor activating and discriminative stimulus effects of nicotine at doses that did not antagonize the locomotor depressant and operant response rate effects of nicotine. Hence, such dissociations based on the behavior measured are not surprising. Similarly, the dose of MLA used in our study was chosen based on the work of Löf et al. (2010) and Guy and Fletcher (2013). Interestingly, at the same dose of 6 mg/kg, Löf et al. (2010) reported that MLA reduced responding in a test of conditioned reinforcement using a sucrose-associated cue, whereas Guy and Fletcher (2013) reported that MLA had no effect on the nicotine-induced enhancement of conditioned reinforcement using a water-associated cue. Hence, this dose allows dissociations to be made based on specifics of the behavioral paradigm. We also note that other research groups have reported behavioral effects of MLA in different paradigms at doses lower than the one we used (Liu, 2014; Wright et al., 2019; Palandri et al., 2021), further validating our use of the 6 mg/kg dose. Nonetheless, testing across a full range of doses for both antagonists would allow for a more complete characterization of the dose-response relationship.

The possibility that other nAChR subtypes (in addition to β2* nAChRs) might also contribute to the nicotine-induced enhancement of conditioned behavior in our study remains plausible. Although DHβE is selective for α4β2 nAChRs, it also binds, although with much less potency, to α9, β4*, and α6β2* nAChRs (Harvey et al., 1996; Verbitsky et al., 2000; Papke et al., 2008; Capelli et al., 2011). However, given that we found no effect of MLA on conditioned behavior in our study, it seems unlikely that α6β2* nAChRs were involved in this behavior. Although MLA is considered selective for α7 nAChR subtypes, it can also bind to α6β2* nAChRs, although with less affinity (Mogg et al., 2002; Capelli et al., 2011). We also note that at the modest dose used in our study, DHβE would preferentially target α4β2 nAChRs, as it is 10–50 fold less potent at other receptor subtypes (Harvey et al., 1996; Verbitsky et al., 2000; Abin-Carriquiry et al., 2006; Papke et al., 2008; Capelli et al., 2011; Soll et al., 2013). Our data thus favors an interpretation of the importance of α4β2 nAChRs in nicotine’s enhancement of conditioned approach triggered by an alcohol cue, with the caveat that other receptor subtypes may still play a role in this behavior. Further testing with other nAChR antagonists may allow for better distinction between the functional roles of different nAChR subtypes.

In a related line of work, Löf et al. (2007) reported that systemic administration of DHβE did not influence an operant response for an ethanol-associated cue (conditioned reinforcement), but intra-VTA infusion of α-conotoxin MII (α-CtxMII) did. As α-CtxMII is a selective antagonist of α3β2* and α6* nAChRs (Cartier et al., 1996; Champtiaux et al., 2002; McIntosh et al., 2004), the work of Löf et al. (2007) thus supports a role for α3β2* and/or α6*, but not α4β2*, nAChRs in the conditioned reinforcing properties of ethanol cues. A noteworthy difference between Löf et al. (2007) and our study is that the former tested the ability of nAChR antagonists to attenuate a behavioral response without other drug treatment whereas the latter tested the ability of nAChR antagonists to attenuate the effect of nicotine on a behavioral response. In other words, the rats in our study received both nicotine (or saline, if in the control group) and the nAChR antagonist during the antagonist test sessions, whereas the rats in Löf et al. (2007) were tested only with the nAChR antagonist. Thus, their aim was to identify specific subtypes of nAChRs that mediate ethanol conditioned reinforcement, whereas our aim was to identify specific subtypes of nAChRs that mediate nicotine’s effect on Pavlovian conditioned responding to an ethanol cue. With this important detail in mind, our findings are consistent with those of Löf et al. (2007). We only found an effect of DHβE in nicotine-treated rats; there was no effect of DHβE on its own in saline-treated rats. This suggests that α4β2* nAChRs are not involved in the normal Pavlovian conditioned approach elicited by an ethanol cue, but rather are involved in the heightened levels of Pavlovian conditioned responding to an ethanol cue brought about by nicotine. The work of Tolu et al. (2017) suggests the pattern observed for appetitive responding just described may also hold for consummatory responding: β2 knockout mice drank similar amounts of alcohol, across a range of concentrations, as wild-type mice, suggesting that β2 nAChRs are not required for normal alcohol intake. Chronic nicotine treatment increased alcohol consumption in wild-type mice, an effect that was absent in β2 knockout mice, suggesting that β2* nAChRs are needed for increased levels of alcohol consumption brought on by nicotine treatment (Tolu et al., 2017). These observations are particularly relevant to pharmacological therapies that may be developed to co-treat alcohol use disorder and nicotine dependence.

Although not the primary aim of our study, we also found that there was no relationship between home cage ethanol consumption and terminal levels of Pavlovian conditioned responding in our sample of rats. This suggests that the processes that act to promote conditioned responses to alcohol cues may be distinct from those that fuel alcohol consumption, an important consideration for clinical relevance. We note, however, that alcohol cues can elicit a variety of Pavlovian conditioned responses (Glautier and Drummond, 1994), and it is possible that a different Pavlovian conditioned response than the one we measured could be correlated with free-choice ethanol consumption in other contexts. Nonetheless, the literature suggests this relationship may not be straightforward, as physiological reactivity to an alcohol cue and craving were positively correlated with each other in a sample of treatment-seeking alcohol-dependent patients, but the magnitude of these measures did not predict relapse (Witteman et al., 2015). Yet, in a sample of social drinkers, there was a significant correlation between the magnitude of alcohol cue reactivity in the lab and the self-reported number of standard drinks consumed per week in the real world (White and Staiger, 1991). The rodent literature also suggests that the relationship between home cage voluntary ethanol consumption and conditioned response measures is complex, with clearer evidence for a consistent correlation of home cage ethanol drinking with operant oral self-administration than with appetitive Pavlovian paradigms, such as conditioned place preference (Green and Grahame, 2008). Thus, factors such as the type of conditioning paradigm and model system must be taken into account when attempting to connect appetitive and consummatory behaviors with respect to alcohol.

We did not measure blood ethanol concentration (BEC) in our study, as our primary interest was appetitive, not consummatory, responding. We acknowledge, however, that receipt of alcohol during the Pavlovian conditioning sessions could induce BEC levels that could then modify Pavlovian conditioned responding. We note that there was little variability in the amount of alcohol consumed during Pavlovian conditioning: all rats were given 0.2 ml of 15% ethanol per trial, for a total of 2.4 ml across the entire 63-min long session (12 trials/session). We inspected liquid cups at the end of each session to ensure the majority of the alcohol was being consumed. The literature also supports the idea that conditioned responding can persist in the face of progressive intoxication, suggesting that rats remain capable of demonstrating Pavlovian conditioned responses even with BECs that ranged from 0–57 mg/dl, with an average of 16 mg/dl (Cofresí et al., 2018). Nonetheless, including BEC levels in future work would allow for further investigation of how BEC may influence appetitive conditioning.

We deliberately chose to focus on the goal-tracking response in our study, as this work was an extension of our previous findings (Maddux and Chaudhri, 2017) and we wished to keep the behavioral paradigm consistent. However, a limitation of the current design is that it did not readily allow for measurement of the sign-tracking response. Given reports of nicotine’s effects on sign-tracking (Palmatier et al., 2013, 2014; Overby et al., 2018; Stringfield et al., 2019; but see Loney et al., 2019; Angelyn et al., 2021), it would be useful to know if such responding is also mediated by the same nAChR subtypes as the enhanced goal-tracking response or if other receptor subtypes are involved in the nicotine-induced enhancement of sign-tracking. Future work in our laboratory will address this possibility. Future work in our laboratory will also address potential sex differences in this behavior. We used only male rats in this initial study, given that male and female rats differ in their sensitivity to nicotine (Donny et al., 2000; Chaudhri et al., 2005; Loney and Meyer, 2019). However, given Stringfield et al.’s (2019) report on sex differences in sign-tracking and a growing body of literature showing sex differences in incentive motivation (Dickson et al., 2015; Barker and Taylor, 2019), the inclusion of females in future work is warranted.

In general, across studies, nicotine has been shown to enhance Pavlovian conditioned responding to a reward cue, although in some studies this is manifested as enhanced goal-tracking (Olausson et al., 2003; Guy and Fletcher, 2013; Maddux and Chaudhri, 2017; Loney et al., 2019; Angelyn et al., 2021), whereas in others, this is manifested as enhanced sign-tracking (Palmatier et al., 2013, 2014; Guy and Fletcher, 2014). Diverse observations across different studies likely result from procedural differences employed by different research groups, as it is known that the characteristics of the conditioned stimulus (CS), such as modality, localizability, and location, influence the form of the conditioned response (Holland, 1977, 1980). Much of the previous work that examined the effect of nicotine on Pavlovian conditioned responding (either goal-tracking or sign-tracking or both) used a sweet natural reward, such as sucrose (Palmatier et al., 2013; Stringfield et al., 2019), a sweetened chocolate solution (Palmatier et al., 2014), or sweetened condensed milk (Overby et al., 2018), as the unconditioned stimulus (US). Other research groups (Olausson et al., 2003; Guy and Fletcher, 2013, 2014) used water, a natural but not sweet reward, as the US. Fewer researchers have explored the effect of nicotine on the Pavlovian conditioned response using a drug reward, such as alcohol, as the US (Maddux and Chaudhri, 2017; Loney et al., 2019; Angelyn et al., 2021). Given that comparisons of results across different laboratories are clouded by procedural differences, a systematic comparison of nicotine’s effects on Pavlovian conditioned responding (goal-tracking and sign-tracking) engendered by natural vs. drug reward cues is needed, although we note that Angelyn et al. (2021) have recently begun this work. Unlike other drugs of abuse, alcohol as a drug reward is particularly attractive for such studies given that it is ingested orally, and can be matched for caloric content with specific concentrations of liquid sucrose solution. Such studies directly comparing nicotine’s effects on the form of the Pavlovian conditioned response (goal-tracking and sign-tracking) to an alcohol cue vs. a sucrose cue are currently underway in our laboratory. Findings from such studies will be instructive in reconciling disparate findings across labs and helping to unify the literature. Such findings will also have translational relevance, as it will be useful to know if there are effects of nicotine that are specific to an alcohol cue, in contrast to a cue for natural reward.

In our study, nicotine enhanced conditioned responding (by definition, greater responding during the CS interval compared to the pre-CS interval) in the paired behavioral training group. However, there was a mild effect of nicotine in the unpaired behavioral training group as well, with nicotine-treated rats showing the opposite pattern: increased liquid cup entries during the pre-CS interval compared to the CS interval. At first glance, this suggests a non-specific effect of nicotine on behavior. Given the known effects of nicotine to increase locomotor activity (Clarke and Kumar, 1983; Stolerman et al., 1995), as well as increase responsivity to/for sensory stimuli (Donny et al., 2003; Perkins and Karelitz, 2014), such possibilities must be considered. We acknowledge that these other processes could have contributed to responding in our paradigm, however, we have evidence of specific effects of nicotine on behavior above and beyond non-specific responding. First, if nicotine increased responsivity to sensory stimuli (such as the audiovisual CS used in this experiment), we would expect to observe a higher response during the CS interval in the nicotine-treated rats regardless of the behavioral training group, but this is not what was found. Rather, nicotine enhanced responding during the CS interval only for rats in the paired behavioral training group. The lack of elevated CS responding in the unpaired behavioral training group suggests that nicotine did not simply enhance responding to sensory stimuli, but rather enhanced responding to sensory stimuli that were made motivationally significant through their association with the alcohol reward US. However, we note that stimulus-induced responding may take different forms: we measured liquid cup entries, but salient stimuli often also elicit an orienting response (Holland, 1977, 1980), which was not measured in this study. Given that nicotine has been shown to increase the orienting response to a visual cue that served as an inhibitory stimulus in a negative occasion setting paradigm (MacLeod et al., 2010), the possibility remains that nicotine could have enhanced other forms of responding to the CS, such as the orienting response, in the unpaired behavioral training group in our experiment. This highlights the need for multiple measures of responding in future work. We note, however, that even if nicotine enhanced the orienting response to the CS in the unpaired group, this may not denote a non-specific effect of nicotine on behavior. That is, in our explicitly unpaired group, the alcohol US never occurs when the CS is present or has recently been presented; in this way, the CS in our paradigm may take on inhibitory properties for the unpaired group. Such a stimulus would still be considered motivationally significant (through its contrasting lack of association with the alcohol reward US) and thus an enhanced orienting response to it may represent a specific effect of nicotine, rather than a general enhancement to non-meaningful sensory stimuli.

Second, a simple locomotor stimulant effect of nicotine as the reason for our results is ruled out by the finding of increased responding during specific behavioral epochs/events. If nicotine simply increased locomotor activity indiscriminately, we would expect to observe more liquid cup entries in nicotine-treated rats compared to saline-treated rats during any interval of time. However, this was not what was found. Rather, nicotine enhanced responding during an interval of time that was meaningful with respect to reward delivery in the two different behavioral training groups. For the paired group, the best cue for reward is the CS; for the explicitly unpaired group, the best “cue” for reward is the *lack* of the CS (such as during the pre-CS interval). We observed a higher response in nicotine-treated groups for both intervals, respectively, in the two behavioral training groups (i.e., higher CS responding in the paired group and higher pre-CS responding in the unpaired group). Such a pattern suggests that nicotine enhanced reward-seeking in a strategic way. Finally, a locomotor account of the data is further ruled out by the observation that nicotine-treated rats never showed greater liquid cup entries compared to saline-treated rats during the 2-min delay period that preceded the start of each training session (Supplementary Figure 1). In fact, nicotine-treated rats made fewer liquid cup entries than saline-treated rats during the 2-minute delay period, an effect largely driven by delay period entries in the first few training sessions, before the nicotine-treated rats had repeated exposure to nicotine and likely reflecting a transient locomotor depressant effect of nicotine in non-tolerant subjects (Clarke and Kumar, 1983; Stolerman et al., 1995).

Although nicotine clearly elevates the conditioned response to a reward-associated cue, additional research is needed to determine if nicotine exerts this effect primarily by influencing learning or performance. That is, nicotine may increase conditioned responding by enhancing the formation of CS-US associations or it may more simply act to increase behavioral output (a more direct effect on the CR). The current study does not allow us to disentangle these possibilities, but some predictions follow from these alternatives. If nicotine enhances the formation of CS-US associations, then the elevated response elicited by the CS would be expected to be observed during later stages of testing (after the CS-US association has already been formed), even in the absence of nicotine. By contrast, if nicotine acts more directly on the performance of the CR to elevate responding, then responding elicited by the CS would be expected to decline if nicotine treatment were discontinued. The literature provides mixed results in this regard: Palmatier et al. (2013) reported continued enhanced sign-tracking in previously nicotine-treated rats who were switched to saline treatment during later stages of testing, however, Guy and Fletcher (2014) reported that increased sign-tracking brought about by nicotine treatment was abolished when nicotine was replaced with saline. These disparate findings may be due to procedural differences between the studies, such as the specific measure of sign-tracking behavior employed, the type of US used, and/or the amount of behavioral training provided under nicotine treatment before saline substitution tests commenced. Future work in our lab will incorporate discontinuation of nicotine to probe learning vs. performance accounts of nicotine-induced enhancement of goal-tracking. Although the current study was not designed to address these possibilities, some speculative hints exist in our current data. For instance, if nicotine enhances learning, then nAChR antagonists at test might be expected to have no effect on an already-learned conditioned response. The fact that the β2* selective nAChR antagonist DHβE attenuated the nicotine-induced enhancement of conditioned responding during test sessions suggests that nicotine may exert this effect via the performance of the CR. However, an intriguing but speculative alternate explanation of the modest residual enhancement (albeit not statistically significant) in the paired nicotine group during DHβE antagonism (see Figure 5A) is that nicotine did influence learning to some degree earlier in training. Yet the observation that conditioned responding did not change across trials in the trial-by-trial analysis of the DHβE test session suggests that a performance-based mechanism is more likely responsible for nicotine’s enhancement of conditioned responding. DHβE attenuated nicotine’s increase of the conditioned response from the very first trial. A learning-based mechanism would be expected to produce a more gradual, within-session reduction in responding. It is conceivable that nicotine could influence both learning and performance in this paradigm, but further work is needed to fully investigate this question. Indeed, the lack of an effect of MLA in our test session must be interpreted with caution with respect to the above logic. The design of our behavioral paradigm (test session that followed many training sessions) favors the ability to detect effects at the level of performance. While the null effect of MLA in this paradigm supports the interpretation that α7 nAChRs are not involved in the performance of the nicotine-induced enhancement of conditioned responding, the possibility remains that they could be involved in this behavior at the level of learning. Future work will use a variety of behavioral paradigms, with testing at different time points, to dissect the contributions of different nAChR subtypes to learning vs. performance of the nicotine-augmented Pavlovian conditioned response.

Even the learning vs. performance distinction is comprised of several further mechanisms that may operate to increase Pavlovian conditioned approach behavior by nicotine. For instance, the idea that nicotine may increase the conditioned response by enhancing the formation of CS-US associations (a learning-based interpretation) is itself composed of component processes that may act on the CS, the US, or both. First, nicotine may increase the attention paid to the CS, and hence make it more associable (Mackintosh, 1975; Pearce and Hall, 1980). In support of this idea, nicotine has been shown to increase the orienting response to a visual stimulus, which is often considered a measure of attentional processing (MacLeod et al., 2010; Meyer et al., 2015, 2016). Alternatively, nicotine may enhance the incentive salience of the CS (King and Meyer, 2022), a proposition supported by findings from a range of behavioral paradigms across different research groups (Olausson et al., 2004; Palmatier et al., 2006, 2007; Caggiula et al., 2009; Overby et al., 2018). This possibility is especially intriguing, as some groups have shown that the enhanced incentive salience of cues returns to control levels once nicotine treatment is stopped (Guy and Fletcher, 2014; Overby et al., 2018), suggesting a potent but short-lived effect of nicotine, which may have significance for eventual clinical applications. Third, nicotine may enhance the reward value of the US, and hence make the US capable of supporting higher levels of conditioned behavior, an idea derived from seminal learning theory (Rescorla and Wagner, 1972). There is some evidence for this idea, in that nicotine increased the breakpoint for alcohol in a progressive ratio task in rats (Frye et al., 2018) as well as humans (Barrett et al., 2006). However, other work suggests that nicotine does not enhance the positive reward value of alcohol across the board, but rather exerts effects in very specific ways (Loney et al., 2018). This conclusion was reached from the observation that nicotine reduced ethanol-induced conditioned taste aversion but not ethanol-induced conditioned place aversion (Loney et al., 2018). The reduction in ethanol-induced conditioned taste aversion by nicotine could be interpreted as nicotine affecting ethanol reinforcement by increasing its reward value (or decreasing its aversive properties), but this interpretation is harder to accept given the lack of nicotine effect in the conditioned place aversion procedure. Hence, the literature remains mixed with respect to the conditions under which nicotine may increase the reward value of an alcohol US. Fourth, nicotine may increase the conditioned response by promoting habitual behavior (Clemens et al., 2014; Loughlin et al., 2017; Luijten et al., 2020). In our paradigm, this would involve nicotine acting upon a stimulus-response association of the CS and the liquid cup entry CR, in contrast to a goal-directed CS-US association. US devaluation procedures could be used to distinguish between these two types of responding (Adams and Dickinson, 1981; Balleine and Dickinson, 1998). Of course, these possibilities are not mutually exclusive, and nicotine may exert its enhancement of Pavlovian conditioned responding through a combination of mechanisms. Future work utilizing sophisticated behavioral paradigms is thus needed to parse these processes.

In our study, we administered nicotine via systemic subcutaneous injections. Hence, we are not able to pinpoint specific brain regions in which nicotine may have acted to exert the behavioral effects we observed. Similarly, as the nAChR antagonist DHβE was also administered systemically, we do not know where in the brain DHβE acted to attenuate the behavioral effects of nicotine. However, a candidate brain area for further testing is the ventral tegmental area (VTA). This suggestion is based on a recent report showing that nicotine’s ability to enhance conditioned reinforcement is based on its actions on α4β2 nAChRs in the VTA (Tabbara and Fletcher, 2019). However, we note that Tabbara and Fletcher (2019) used water, a natural, non-drug reward, as the US during the Pavlovian conditioning phase of their study, and hence animals responded for a water-associated cue during the tests of conditioned reinforcement. It remains to be determined if similar mechanisms are at play for drug-associated cues, although previous findings suggest cholinergic involvement in the VTA to mediate drug cue-directed behavior (Laviolette and van der Kooy, 2003; Zhou et al., 2007; Solecki et al., 2013; Nunes et al., 2019). Finally, although there is strong support that nicotine’s neurochemical and behavioral effects are centrally mediated (Reavill and Stolerman, 1990; Corrigall et al., 1994; Nisell et al., 1994a, b; Picciotto and Corrigall, 2002), potential peripheral effects of nicotine in our paradigm must also be acknowledged, given our route of drug administration (Kiyatkin, 2014). Future work that targets specific brain structures will allow central vs. peripheral effects of nicotine to be disentangled.

Our results reported here add to a substantial body of literature that shows that nicotine increases conditioned responding to reward-associated cues (Olausson et al., 2003; Guy and Fletcher, 2013, 2014; Palmatier et al., 2013, 2014; Stringfield et al., 2018, 2019), and extends previous work (Maddux and Chaudhri, 2017; Loney et al., 2019; Angelyn et al., 2021) to show that nicotine’s enhancement of conditioned responding to an alcohol-associated cue is mediated through β2* nAChRs. Future work will localize brain targets that are the loci of these effects, as well as further probe the effect of nicotine on the development of different forms of Pavlovian conditioned responding and explore potential sex differences in this paradigm. Moreover, the degree to which nicotine’s amplifying effect is specific to an alcohol cue, compared to a cue for a non-drug reward, will be investigated, along with the behavioral and neurobiological mechanisms responsible for these effects. Given the co-use of nicotine and alcohol (DiFranza and Guerrera, 1990; Dani and Harris, 2005; Cross et al., 2017), such findings may help inform substance abuse treatment strategies. The challenge remains to identify pharmacological treatments that specifically affect drug-related behaviors, while sparing natural reward-related behaviors, before preclinical findings may be best translated into the clinical context.

## Data Availability Statement

The raw data supporting the conclusions of this article will be made available by the authors, without undue reservation.

## Ethics Statement

The animal study was reviewed and approved by Lake Forest College Institutional Animal Care and Use Committee.

## Author Contributions

J-MM formulated overarching research goals and wrote the manuscript. J-MM, LG, and NK participated in study design and methodology, conducted behavioral experiments, and performed statistical analyses. All authors contributed to the article and approved the submitted version.

## Funding

This work was supported by an equipment grant from the Sherman Fairchild Foundation, the Department of Psychology of Lake Forest College, and start-up funds provided to J-MM by Lake Forest College.
