# Supplementary material for: β2* nicotinic acetylcholine receptor subtypes mediate nicotine-induced enhancement of Pavlovian conditioned responding to an alcohol cue
Source: Front Behav Neurosci. 2022 Oct 12;16:1004368. doi: 10.3389/fnbeh.2022.1004368 (PMC9596985; doi:10.3389/fnbeh.2022.1004368)
Supplement: Supplementary file 1 [file Data_Sheet_1.pdf]

Supplementary Material

1 Supplementary Figures and Tables

1.1 Supplementary Figures

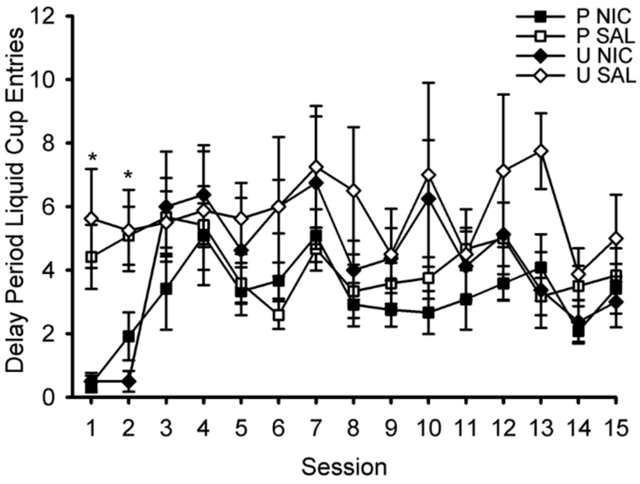

Supplementary Figure 1. Nicotine-treated rats made fewer liquid cup entries than their saline-treated counterparts during the 2 min delay period before house light onset at the start of each session. Data represent the mean (±SEM) number of liquid cup entries per session during the delay period. Black symbols indicate nicotine (NIC) treatment and white symbols indicate saline (SAL) treatment. Square symbols indicate the paired behavior group (P) and diamond symbols indicate the unpaired behavior group (U). ANOVA with the within-subject factor of session and the between-subject factors of behavior group and drug group indicated that delay period liquid cup entries varied across sessions [main effect of session: $F(14,504) = 3.705$, $p < 0.001$]; the paired behavior group made fewer delay period liquid cup entries than the unpaired behavior group [main effect of behavior group: $F(1,36) = 5.057$, $p = 0.031$]; nicotine-treated rats made fewer delay period liquid cup entries than saline-treated rats [main effect of drug group: $F(1,36) = 4.517$, $p = 0.040$]; and the effect of drug group was most pronounced in early training sessions [interaction of session x drug group: $F(14,504) = 1.942$, $p = 0.029$]. Follow-up independent samples t-tests on data collapsed across behavior group showed that nicotine-treated rats made fewer delay period liquid cup entries than saline-treated rats in sessions 1 [$t(38) = -5.087$, $p <0.001$] and 2 [$t(38) = -4.327$, $p < 0.001$]. *$p < 0.05$, NIC < SAL

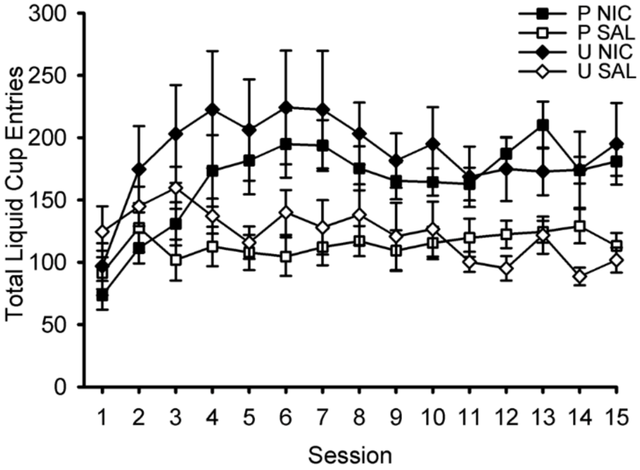

Supplementary Figure 2. Total liquid cup entries made per session during the Pavlovian conditioning phase of the experiment. Nicotine-treated rats made more total liquid cup entries than their saline-treated counterparts. Data represent mean (±SEM) number of liquid cup entries per session. Black symbols indicate nicotine (NIC) treatment and white symbols indicate saline (SAL) treatment. Square symbols indicate the paired behavior group (P) and diamond symbols indicate the unpaired behavior group (U). ANOVA with the within-subject factor of session (1-15) and the between-subjects factors of behavior group (paired, unpaired) and drug group (nicotine, saline) revealed a main effect of session, $F(14, 504) = 5.285$, $p < 0.001$, a main effect of drug group, $F(1,36) = 14.056$, $p = 0.001$, and interactions of session x drug group, $F(14,504) = 4.476$, $p < 0.001$, and session x behavior group, $F(14,504) = 2.929$, $p = 0.008$. Importantly, there was no significant main effect of behavior group [$F(1,36) = 1.023$, $p = 0.319$], demonstrating that the paired and unpaired training groups showed similar total numbers of liquid cup entries overall. Moreover, follow-up post-hoc testing of the significant session x behavior group interaction showed that the two groups had significantly different numbers of total liquid cup entries in only one session, Session 3, $t(38) = -2.849$, $p = 0.007$, with the unpaired group making more liquid cup entries than the paired group in that session.

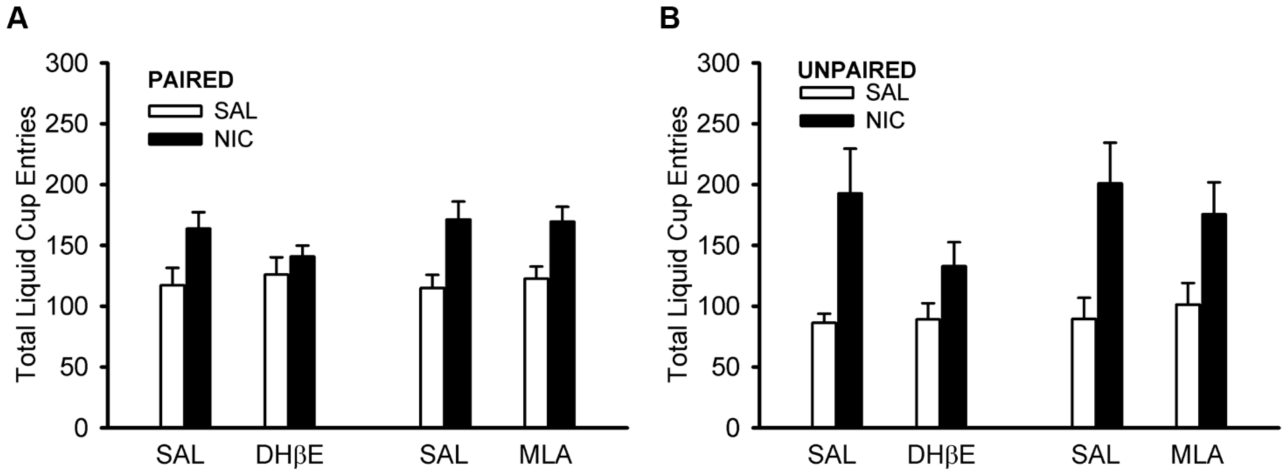

Supplementary Figure 3. Total liquid cup entries made per session during the nAChR antagonist test sessions. The β2* nAChR antagonist dihydro-beta-erythroidine (DHβE) reduced the nicotine-induced increase in total liquid cup entries, whereas the α7 nAChR antagonist methyllycaconitine (MLA) did not. Data represent the mean (± SEM) number of liquid cup entries per session for the paired **(A)** and unpaired **(B)** behavioral training groups. Black bars denote nicotine (NIC) drug group and white bars denote saline (SAL) drug group. For the DHβE test session, ANOVA with the within-subject factor of nAChR antagonist treatment (DHβE, saline) and the between-subject factors of behavior group (paired, unpaired) and drug group (nicotine, saline) showed a main effect of drug group, $F(1,36) = 15.339$, $p < 0.001$, and an interaction of nAChR antagonist treatment x drug group, $F(1,36) = 5.372$, $p = 0.026$. This interaction denotes that the difference in total liquid cup entries between the nicotine and saline drug groups was attenuated when DHβE was administered, compared to saline. For the MLA test session, ANOVA with the within-subject factor of nAChR antagonist treatment (MLA, saline) and the between-subject factors of behavior group (paired, unpaired) and drug group (nicotine, saline) showed only a main effect of drug group, $F(1,36) = 19.787$, $p < 0.001$. There was no effect of, nor interactions with, nAChR antagonist treatment in the analysis of total liquid cup entries when MLA was administered.

1.2 Supplementary Tables

Supplementary Table 1.

Statistical Results of Omnibus 4-way ANOVA for Pavlovian Conditioned Approach Data during Pavlovian Conditioning Phase

**Main effects**	**df_1, df_2**	**F**	***p***
Session	14, 504	16.059	< 0.001*
Interval	1, 36	44.805	< 0.001*
Behavior Group	1, 36	34.197	< 0.001*
Drug Group	1, 36	6.861	0.013*
**Interactions**	**df_1, df_2**	**F**	***p***
Behavior Group x Drug Group	1, 36	4.471	0.041*
Session x Behavior Group	14, 504	18.566	< 0.001*
Session x Drug Group	14, 504	2.262	0.056
Interval x Behavior Group	1, 36	48.039	< 0.001*
Interval x Drug Group	1, 36	3.561	0.067
Interval x Session	14, 504	20.261	< 0.001*
Session x Behavior Group x Drug Group	14, 504	1.632	0.160

**Interactions**	**df_1, df_2**	**F**	***p***
Interval x Session x Behavior Group	14, 504	20.615	< 0.001*
Interval x Session x Drug Group	14, 504	1.501	0.200
Interval x Behavior Group x Drug Group	1, 36	6.008	0.019*
Interval x Session x Behavior Group x Drug Group	14, 504	1.811	0.123

Note. *indicates statistical significance assessed against an alpha level of 0.05. The Huynh-Feldt correction has been used for violations of sphericity.
